# Plasma Amino Acid Appearance and Status of Appetite Following a Single Meal of Red Meat or a Plant-Based Meat Analog: A Randomized Crossover Clinical Trial

**DOI:** 10.1093/cdn/nzac082

**Published:** 2022-05-04

**Authors:** Toan Pham, Scott Knowles, Emma Bermingham, Julie Brown, Rina Hannaford, David Cameron-Smith, Andrea Braakhuis

**Affiliations:** Discipline of Nutrition, School of Medical Sciences, Faculty of Medical and Health Sciences, The University of Auckland, Auckland, New Zealand; Auckland Bioengineering Institute, The University of Auckland, Auckland, New Zealand; Smart Foods Innovation Centre of Excellence, AgResearch Ltd, Palmerston North, New Zealand; Smart Foods Innovation Centre of Excellence, AgResearch Ltd, Palmerston North, New Zealand; Discipline of Nutrition, School of Medical Sciences, Faculty of Medical and Health Sciences, The University of Auckland, Auckland, New Zealand; Bioinformatics and Statistics Team, AgResearch Ltd, Palmerston North, New Zealand; College of Engineering, Science and Environment, The University of Newcastle, Newcastle, Australia; College of Health, Medicine and Wellbeing, The University of Newcastle, Newcastle, Australia; Discipline of Nutrition, School of Medical Sciences, Faculty of Medical and Health Sciences, The University of Auckland, Auckland, New Zealand

**Keywords:** protein, amino acid, red meat, plant-based meat analog, meat alternative

## Abstract

**Background:**

Red meat is a nutrient-dense food and a dietary staple. A new generation of plant-based meat analogs (PBMAs) have been designed to mimic the experience of eating meat, but there is limited evidence about their digestive efficacy and nutritional quality.

**Objectives:**

We compared the postprandial digestive response of a single meal containing meat commercially raised in New Zealand, including lamb, on-farm pasture-raised beef (Pasture), or grain-finished beef (Grain) with a PBMA (Beyond Burger; Beyond Meat) sold through consumer retail. The primary outcome was the appearance of amino acids in plasma. Secondary outcomes included glucose and insulin, appetite assessment, and anthropometry.

**Methods:**

Thirty healthy men (20–34 y) participated in a double-blinded randomized crossover trial. Each consumed 1 of the 4 test meals on 4 occasions separated by a washout period of at least 1 wk, following an overnight fast. The meal was a burrito-style wrap containing meat or PBMAs, vegetables, salsa, and seasonings in a flour tortilla. The amount of Pasture, Grain, Lamb, or BB was 220 g raw (∼160 g cooked). Venous blood samples were collected over 4 h. Appetite and hunger status was scored with visual analog scales.

**Results:**

Pre-meal amino acid concentrations in plasma did not differ by group (*P *> 0.9), although several nonessential amino acids differed strongly according to participant BMI. Postprandial amino acids peaked at 2–3 h in all groups. The BB meal produced significantly lower plasma concentrations of total, essential, branched-chain, and non-proteogenic amino acids than the Lamb, Pasture, or Grain meals, based on AUC. There were no significant differences between meal groups in scores for hunger, fullness, or cravings.

**Conclusions:**

Red meat meals exhibited greater bioavailability of amino acids compared with the PBMA (BB). Pasture versus Grain origins of the beef had little influence on participants’ responses. This trial was registered at ClinicalTrials.gov as NCT04545398.

## Teaser

This randomized crossover clinical trial investigated plasma amino acid appearance and digestive responses following a single mixed meal containing red meat or a plant-based meat alternative.

## Introduction

A consumer trend to reduce meat intake has spurred the development and availability of alternative products. A new generation of highly refined, plant-based meat analogs (PBMAs) is designed to mimic the taste, texture, and presentation of meat (1), providing a way to moderate meat consumption with only minimal change in dietary habits. What were once niche foods aimed at vegetarians are increasingly marketed to omnivores and flexitarians (2). This product category is perceived to have advantages in its environmental footprint, sustainability, animal welfare, and allied consumer perceptions (3–5). However, evidence of digestive efficacy, nutritional quality, and health benefits is still scarce.

Meat is a nutrient-dense whole food and a dietary staple for many cultures. It contains proteins and peptides, long-chain fatty acids, and complex lipids, vitamins, minerals, and additional micronutrients that are otherwise difficult to obtain (6). A wide variety of other constituents and metabolites found in meat, many of which are not listed on traditional nutrient information panels, also have potential health implications (7). Nutritional differences in protein content and quality between red meat and PBMAs have not been thoroughly assessed, nor has consideration been given to PBMAs in the context of a meal or the background diet of the consumer.

Beef cattle raised under pasture or grain-finished systems may have differences in metabolic efficiency and meat quality (8). Meat from ruminant animals raised on predominantly pasture-based diets has higher concentrations of PUFAs and related lipids derived from leaf oils (i.e., green grass) compared with meat from grain-finished animals (9). With regard to protein digestion and metabolism, differences in gene expression within cattle raised on grass or grain have shown altered metabolic pathways and content of metabolites in the liver. Given the importance of the liver in protein metabolism, differences in liver content and function may influence the nutrition composition of the meat later consumed by humans. In the grass-fed and grain-finished cattle, the altered gene expression was mainly responsible for lowering intramuscular fat, cholesterol, and yellow carcass-fat meat in grass-fed cattle and hence altered the nutrition composition of beef (10). However, the implications of consuming grass versus grain-finished beef, lamb, and PBMAs on the nutrition composition of the test meals and the resultant amino acid (AA) profile post–human consumption are, in theory, very important, but the clinical relevance is equally so. The AA profile of beef and lamb has previously been reported in cooked and uncooked samples (11), in which the AA profiles of lamb and beef were comparable, with lamb having a lower histidine content. Few investigations have focused on how the composition of the meat relates to human digestion and absorption in healthy individuals.

The labeled content of total protein in meat and PBMAs can appear comparable; however, the quality of proteins and balance of AAs may result in different postprandial responses (12, 13). Certain AAs, particularly leucine, alter muscle protein synthetic rates. In previous research conducted in middle-aged men, when 24 g soy protein was compared with an isonitrogenous amount of beef protein, soy protein induced less muscle synthesis both at rest and following resistance exercise (14). As such, the AA profile, not simply total protein, may influence the nutritional value of meat or its alternative.

As an ultra-processed food, the nutrient composition of PBMAs is achieved through sophisticated formulations of ingredients, extracts, and additives. These may be far removed from their plant origins, potentially devoid of concomitant nutrients and naturally present phytochemicals, and changed in their structural characteristics (15, 16). Such manipulations may have nutritional consequences. For example, a controlled-feeding study recently reported that consuming diets of ultra-processed food led to excess energy intake and weight gain, and may exacerbate metabolic syndrome (17). Increased BMI in overweight men has been shown to influence postprandial glycemic response compared with lean adults (18).

While descriptions of the macronutrient and some micronutrient values of beef, lamb, and PBMAs have been published (8, 19–22), the impact on plasma AA appearance following protein ingestion remains to be established. Accordingly, we aimed to measure the postprandial concentrations of AAs in the plasma of healthy young men who consumed 4 standardized meals that differed in protein source as either pasture-raised or grain-finished beef, lamb, or PBMAs. We also compared the meals’ effect on self-reported scores of appetite, hunger, and fullness. We hypothesized that the PBMA meal would exhibit a lower bioavailability of AAs in circulated blood compared with other meat meals and were interested in characterizing the response to different red meat types.

## Methods

### Participant recruitment

Thirty healthy men (20–34 y) were recruited via social media advertising in Auckland, New Zealand. All were omnivores willing to consume red meat and PBMAs for the trial. Exclusion criteria were chronic health conditions, hyperlipidemia, obesity [BMI (kg/m^2^) ≥30], use of medications (except occasional use of nonsteroidal anti-inflammatory drugs and antihistamines), history of anosmia and ageusia (issues with taste and smell), current dieting or disordered eating pattern, and smoking tobacco or recreational drugs. Also excluded were those with a Three-Factor Questionnaire-R18 (23, 24) score greater than 75%, implying that their perception of food may be influenced by underlying psychological issues, with the cutoff deemed clinically relevant by the dietetic team. Enrolled participants gave written informed consent to authorize all future uses for their data in published research. Participants received a gift voucher to reimburse their time and efforts for study completion. The information or samples collected in this study were kept for a total of 10 y. Any data results outside of the normal healthy range were informed to participants and subsequent follow-up with their usual doctors could be arranged if appropriate. The Principal Investigator was responsible for the security of identifiable data and circulated the findings of this study to participants.

The trial was approved by the New Zealand Ministry of Health's Health and Disability Ethics Committees (19/STH/226) and conducted in accordance with ethical standards from the 1964 Declaration of Helsinki. It was registered with a Universal Trial Number (U1111-1244-9426) and at ClinicalTrials.gov (NCT04545398). The trial was conducted between October and December 2020 at the Clinical Research Centre of the University of Auckland, New Zealand.

### Trial design

A double-blinded randomized crossover design was used to compare postprandial responses to a breakfast meal. Each participant consumed 1 of 4 test meals on 4 occasions separated by a washout period of at least 1 wk. Meals were provided in random order based on a computer-generated sequence. Research staff preparing and serving the meals were different from those collecting data, and the meal serving area was in a different location from where the anthropometry, phlebotomy, and questionnaires were completed. The meat and PBMA raw materials were parceled into generic packaging and then labeled with a code that designated their intended meal type. Staff preparing the meals and participants consuming them were blinded to the key. All meat was also minced to maximize protein absorption and mimic the format of the BB product as well as disguise the protein type to participants.

### Treatments

The test meals contained either pasture-raised beef (Pasture), grain-finished beef (Grain), pasture-raised lamb (Lamb), or Beyond Burger™ (Beyond Meat), a plant-based meat analog (hereafter BB, when referring specifically to the treatment group). The beef was differentiated by its on-farm production system. The Pasture was from Angus steers grazed on free-range pastures of predominantly ryegrass and white clover. Grain was from Angus steers grazed on pastures then finished in feedlots for an average of 122 d on a ration of maize silage, barley, wheat, and straw. Livestock class, age, and weight were similar. The meat marbling score [3 to 4 out of 9 on the Australian Grading System (25)] and intrinsic pH (average: 5.5) of the beef was consistent between production systems. The pasture-raised lamb was also sourced from free-range New Zealand farms.

In all cases, the cut of meat used was the full tenderloin [*musculus (M.) psoasmajor *+* M. psoas minor *+* M. iliacus*]. This is typically removed in 1 piece from the full rump and loin. To accommodate between-animal variation, tenderloins were collected from 12 Pasture steers, 15 Grain steers, and 40 lambs. The intact meat was aged for at least 21 d at –1.5°C. In preparation for the trial, the Grain tenderloins were trimmed of excess fat, ground together to a homogenous 4-mm mince, then vacuum packed in 500-g aliquots and frozen until needed for the trial. The Pasture tenderloins were not trimmed and so included intermuscular fat. They were processed as per Grain. The Lamb was closely trimmed of fat and similarly processed.

By definition, PBMAs are plant-based foods that mimic the appearance, flavor, and fibrous texture of meat (26). The range of products in this category is diverse and dynamic. For the current trial, we chose Beyond Burger mince, a commercial product based on pea protein, canola oil, and coconut oil. In 2020 it was the closest match to our selection criteria, as follows: *1*) nutrition approximates beef with regard to total energy, *2*) appearance approximates beef, and *3*) readily available to consumers in New Zealand. A sufficient quantity of 1 batch was purchased locally and kept frozen until needed.

### Meal preparation

The test meal was a burrito-style wrap containing meat or BB, fresh and canned vegetables, tomato salsa, and seasonings in a flour tortilla. The amount of Pasture, Grain, Lamb, or BB was 220 g raw (∼160 g cooked). All nonmeat ingredients per meal, including brown onion (53 g), red capsicum (72 g), corn kernels (137 g), tortillas, salsa, seasoning salt, black pepper, and brown sugar, were purchased at local supermarkets. On each testing occasion, meals were cooked fresh according to a standardized recipe and served hot (70°C; Solo probe thermometer; PUREQ). Melted fat was retained with the meal. The weight of the meal as served was approximately 470 g. Alongside meals for participants, additional meals were prepared and frozen for subsequent chemical analysis.

### Trial procedure

The clinical setting had a maximum capacity of 6 people, so we randomly allocated all participants to manageable subsets. The entire trial comprising 4 visits proceeded over 2 mo, with each visit at least 1 wk and no more than 1 mo apart. Participants were asked to maintain their usual lifestyle and physical activity patterns, with a stipulation to fast with only water the night before each visit. Text messages were sent to participants as a reminder. Visits began at 07:30 with measurement of height, weight, and blood pressure (in triplicate using a HEM-7130 digital sphygmomanometer; Omron Healthcare). A forearm antecubital vein was cannulated, and blood samples were collected into vacutainer EDTA blood collection tubes prior to the test meal (Pre) and 4 times postprandially (1, 2, 3, and 4 h). Participants consumed the breakfast meal within 15 min.

At each visit, participants completed a 24-h dietary recall of their prior food and drink consumption, facilitated by an online automated self-administered system (ASA24-Australia-2016; NIH and US Department of Health and Human Services). The ASA24® provides estimates of energy and macro- and micronutrients and has been validated in adult populations (27). The recalls were checked for completeness and cleaned according to ASA24 guidelines including correction of known database issues. A criterion to exclude inaccurate food records was calculated from SDs generated for this population based on the agreement between reported energy intake and predicted total energy expenditure, using a cutoff of 2 SDs (28).

Status of appetite was assessed during each visit using an online visual analog scale questionnaire (Qualtrics; SAP) that has been validated for single-meal investigations (29). Hunger, satisfaction, fullness, and desire to consume something sweet, salty, savory, or fatty were evaluated. Scores were recorded before the meal (Pre), immediately after the meal (0 h), and at 0.5, 1, 2, 3, and 4 h.

A short Qualtrics survey was sent to all participants following each test day to enquire whether they experienced any adverse or side effects.

### Analysis of blood plasma

Blood samples were centrifuged immediately at 1500 × *g* for 15 min at 4°C. Plasma was placed into aliquots and stored at –80°C for subsequent analysis. AAs were measured using ultra-performance liquid chromatography (UPLC), as described previously (30). Briefly, 20 μL of plasma with L-norvaline as internal standard was acid extracted, centrifuged at 14,000 × *g* for 10 min at 4°C, and the supernatant collected. AccQ-Tag reagent (Waters Corp) was then added. UPLC used a Dionex UltiMate™ 3000 system (ThermoFisher Scientific) with a Kinetex separation column preceded by a Krudkatcher inline filter (Phenomenex). Data were captured by Chromeleon 7.1 software (ThermoFisher Scientific). Standard curves for each AA acid were used to calculate plasma concentrations.

Plasma concentrations of insulin and glucose were measured using a Cobas E411 autoanalyzer for immunoassay tests and a Cobas C311 analyzer for clinical chemistry (Roche Diagnostics).

### Analysis of meals

The composition of a representative sample of each meal type was analyzed by an International Accreditation New Zealand–accredited commercial service (Massey Nutrition Laboratory, Palmerston North, NZ). Measurement of moisture used a convection oven at 105°C (AOAC 950.46B) and ash used a furnace at 550°C (AOAC 920.153,923.03). Total protein was determined by the Dumas method applying a conversion factor of 6.25 (AOAC 968.06). Concentrations of the acid-stable AAs were measured using published procedures (31, 32) based on AOAC 994.12. Total fat was determined by Soxtec (AOAC 991.36) or Mojonnier methods (AOAC 922.06). Fatty acid concentrations were measured using Gas Chromatography with Flame Ionization Detection based on Sukhija and Palmquist (33). Total dietary fiber was estimated by the enzymatic Megazyme method (AOAC 991.43). Total sugar measurement used a phenol sulfuric procedure. Minerals were measured using inductively coupled plasma–optical emission spectrometry (ICP-OES). Cholesterol was determined by AOAC 933.08, 970.50, or 970.51.

### Data analysis and statistics

The BMI of each participant was calculated as kg/m^2^ and designated as either normal if ≤25 or overweight if >25. Individual AAs were pooled into classes of essential AAs (EAAs) and their subset branched-chain AAs (BCAAs), nonessential AAs (NEAAs), non-proteinogenic AAs (NPAAs), and total AAs (TAAs).

The Pre (baseline) concentrations of individual plasma AAs were checked for differences attributable to meal type or BMI status using multivariate ANOVA. Pre concentrations of the classes of AAs, appetite scores, plasma glucose, and insulin responses were similarly assessed using a univariate ANOVA.

For plasma amino acids, the incremental AUC of the time-series data was calculated by the trapezoid method after adjusting for Pre, the initial fasting value. An AUC was not determined if data at any of 5 time points were missing or zero. We then fitted a mixed-effects model to the individual AUC values with fixed effects of AA, meal type, and their interaction, and participant ID as a random effect.

The effects of meal type and time on individual and classes of AAs, appetite scores, and glucose and insulin values were evaluated using a repeated-measures ANOVA with time treated as a factor. This allowed estimated marginal means (least-squares means) to be calculated. Tukey-adjusted pairwise comparisons were then carried out to identify statistically significant differences between the means of the meal types at each postprandial timepoint.

Data were analyzed using statistical packages of R software (R Core Team, version 4.1.2,2021). Statistical significance was set at *P* < 0.05. For time-series responses, an effect of meal type was declared only if the meal × time interaction was significant. Results are presented as means ± SEMs unless stated otherwise. Figures were generated with GraphPad Prism (version 7; GraphPad Software, Inc.).

Sample-size calculation was not performed for the AA outcomes of this study. The number of participants (*n* = 30) was considered to be at least as strong and well in excess of the number used in similar research investigating the postprandial response to meat meals, which ranged from 10 participants (34) to 22 participants (35), with an allowance for potential dropouts.

## Results

**FIGURE 1:**
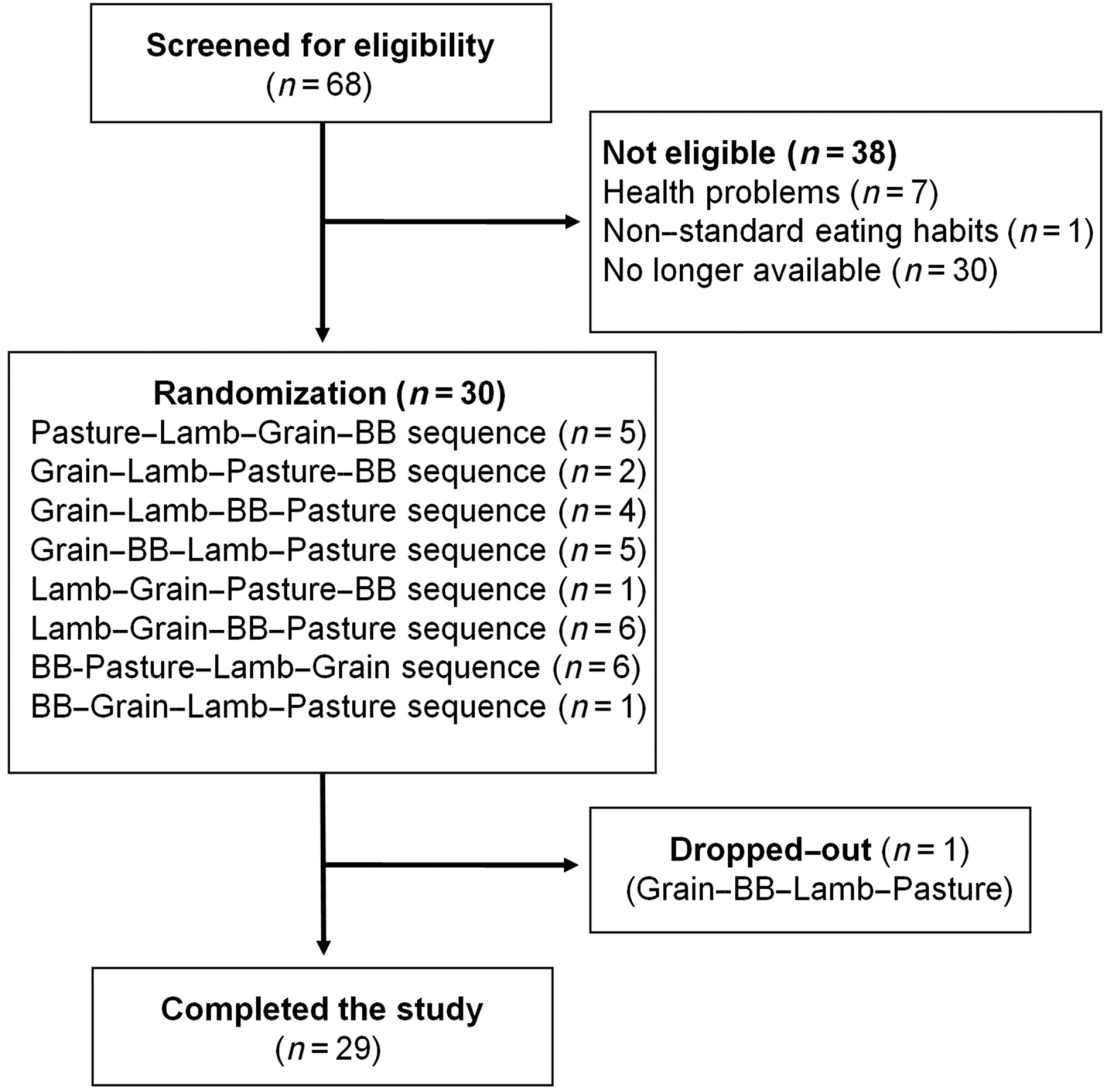
Flowchart of the study participant recruitment. The test meal groups contained either pasture-raised beef (Pasture), grain-finished beef (Grain), pasture-raised lamb (Lamb), or Beyond Burger (BB; Beyond Meat).

**Figure 1** outlines the screening, enrollment, and allocation of participants. Thirty participants were recruited, one dropped out halfway due to work commitments, resulting in 29 consuming all 4 test meals. Postprandial blood collections were complete, except for 1 participant on 1 visit when samples could not be collected at 3 h and 4 h.

The body weight of the participants remained stable over the trial, with little change between visit 1 and visit 4 (0.053 ± 1.3 kg; range: –3.5 to 2.4 kg; *P *= 0.41). Calculated BMI was >25 for 9 of 29 participants (mean: 27.8 vs. 23.1; *P *< 0.001). **Table 1** shows mean physical characteristics, eating behavior scores, and plasma AA concentrations measured on the occasion of the participants’ first clinic visit. No adverse events related to the test meals and phlebotomy procedures were reported. **Table 2** shows participants’ dietary intake on the day prior to their visits. There were no significant differences in energy, protein, total fat, carbohydrates, or fiber between the days before each visit.

**TABLE 1:** Participant characteristics and plasma amino acid concentrations measured before the meal during their first visit^1^

Characteristics	Values
Anthropometry	
Age, y	28.0 ± 3.8
Body weight, kg	76.5 ± 10.1
Body height, cm	176.4 ± 6.0
BMI, kg/m^2^	24.5 ± 2.7
Systolic pressure, mm Hg	118.2 ± 11.8
Diastolic pressure, mm Hg	77.7 ± 9.1
Heart rate, bpm	66.3 ± 9.1
Three-Factor Eating Questionnaire scores, %	
Cognitive restraint	50.9 ± 16.7
Uncontrolled eating	54.2 ± 13.2
Emotional eating	52.6 ± 18.2
BCAAs, µmol/L	
Isoleucine	79.9 ± 11.2
Leucine	142.2 ± 16.3
Valine	261.8 ± 33.2
Other EAAs, µmol/L	
Histidine	50.5 ± 9.4
Lysine	140.3 ± 25.7
Methionine	27.1 ± 3.67
Phenylalanine	73.4 ± 6.6
Threonine	129.9 ± 19.6
Tryptophan	71.0 ± 12.5
NEAAs, µmol/L	
Alanine	356.1 ± 69.3
Arginine	55.1 ± 10.1
Asparagine	55.1 ± 8.1
Aspartic acid	4.2 ± 1.2
Glutamic acid	37.5 ± 13.3
Glutamine	667.3 ± 72.0
Glycine	252.7 ± 45.5
Proline	234.5 ± 70.5
Serine	119.6 ± 17.6
Tyrosine	69.2 ± 10.4
NPAAs, µmol/L	
Citrulline	35.8 ± 5.8
Hydroxyproline	14.3 ± 5.6
Ornithine	40.6 ± 10.4
Taurine	67.0 ± 12.1
TAAs, µmol/L	2985 ± 252

1 Values are means ± SDs; *n *= 29. BCAA, branched-chain amino acid; bpm, beats per minute; EAA, essential amino acid; NEAA, nonessential amino acid; NPAA, non-proteogenic amino acid; TAA, total amino acid.

**TABLE 2:** Estimated nutrient composition of participants’ dietary intake the day before the clinic visit when a particular meal was consumed, based on 24-h dietary recall^1^

Nutrient	Pasture	Grain	Lamb	BB
Energy, kJ	9920 ± 4690	9956 ± 3910	9932 ± 3780	9644 ± 4740
Protein, g	118 ± 49	117 ± 44	118 ± 46	117 ± 59
Total fat, g	103 ± 70	102 ± 73	92 ± 43	96 ± 49
Carbohydrates, g	232 ± 116	238 ± 95	248 ± 124	230 ± 133
Fiber, g	23 ± 12	24 ± 12	24 ± 12	21 ± 11

1 Values are means ± SDs. The test meal groups contained either pasture-raised beef (Pasture), grain-finished beef (Grain), pasture-raised lamb (Lamb), or BB. BB, Beyond Burger (Beyond Meat).

### Nutritional evaluation of test meals

The test meals were designed to be matched for fat and protein content. Variations in the composition of tenderloins collected from cattle and sheep affected the composition of the meals. As shown in **Table 3**, the fat content of the raw minced meats was 17.7%, 9.1%, and 2.4% for the pasture-raised beef, grain-finished beef, and lamb, respectively. The former reflected intermuscular fat included in the mince, which augmented the otherwise lean muscle meat. Protein content was 18.7%, 18.4%, and 21.4%. The fat and protein content of the raw PBMAs was 17.8% and 18.7%, compared with label claims of 15.9% and 17.7%. The crude protein concentrations of all 4 cooked meals were similar (11.2 ± 0.9 g/100 g) and each meal provided approximately 48, 53, 58, and 50 g of total protein.

**TABLE 3:** Nutrient composition of the raw meats in their minced forms, the PBMA as commercially packaged, and the cooked meals (units per 100 g, 470 g per meal)^1^

Nutrient	Pasture	Grain	Lamb	BB
Raw meats and PBMA, g				
Crude protein	18.7	18.4	21.4	18.7
Fat	17.7	9.1	2.4	17.8
Cooked meal				
Crude protein, g	10.3	11.2	12.4	10.7
Fat, g	11.1	6.7	4.3	10.1
Carbohydrates, g	18.1	18.4	19.1	18.3
Total dietary fiber, g	1.6	1.1	1.7	1.9
Sugars, g	3.5	4.0	4.0	3.8
Sodium, g	0.3	0.3	0.3	0.4
Iron, mg	<2.0	<2.0	<2.0	1.9
Zinc, mg	1.2	1.4	1.2	1.1
Cholesterol, mg	27.9	26.0	27.4	<0.5

1 The test meal groups contained either pasture-raised beef (Pasture), grain-finished beef (Grain), pasture-raised lamb (Lamb), or BB. BB, Beyond Burger (Beyond Meat); PBMA, plant-based meat analog.

The AA composition of the meals is shown in **Table 4**. Glu, Asp, and Leu were the most abundant in all meals, and His, Met, and Tau were the least abundant. The greatest proportional difference between meals was for Tau, Met, and Gly. TAA content was greatest in Lamb (11.0%), with little difference among the other meals (9.6% ± 0.16%). EAA content was greatest in Lamb and least in BB. Only minor differences between meals were found for BCAAs and NEAAs.

**TABLE 4:** Amino acid composition of the cooked meals (mg per 100 mg, 470 g per meal)^1^

Amino acids	Pasture	Grain	Lamb	BB
EAAs				
Isoleucine	0.40	0.42	0.49	0.40
Leucine	0.75	0.78	0.89	0.74
Valine	0.49	0.50	0.58	0.51
Histidine	0.28	0.28	0.33	0.23
Lysine	0.66	0.72	0.80	0.53
Methionine	0.27	0.28	0.32	0.18
Phenylalanine	0.44	0.46	0.52	0.53
Threonine	0.39	0.40	0.47	0.33
NEAAs				
Alanine	0.52	0.50	0.59	0.39
Arginine	0.57	0.57	0.65	0.65
Aspartic acid	0.86	0.89	1.01	0.98
Glutamic acid	2.04	2.12	2.38	2.13
Glycine	0.53	0.42	0.47	0.34
Proline	0.66	0.61	0.70	0.66
Serine	0.37	0.38	0.41	0.41
Tyrosine	0.34	0.35	0.40	0.38
BCAAs	1.64	1.70	1.96	1.65
EAAs	3.68	3.85	4.41	3.44
NEAAs	5.89	5.85	6.62	5.94
NPAAs	n/a	n/a	n/a	n/a
TAAs	9.57	9.70	11.03	9.38

1 NPAA was not available in this analysis. The test meal groups contained either pasture-raised beef (Pasture), grain-finished beef (Grain), pasture-raised lamb (Lamb), or BB. BB, Beyond Burger (Beyond Meat); BCAA, branched-chain amino acid; EAA, essential amino acid; NEAA, nonessential amino acid; NPAA, non-proteogenic amino acid; TAA, total amino acid.

### Plasma AA response

The Pre concentrations of AAs (mean of measurements made prior to eating on all visits) did not differ by meal group (*P *= 0.99) but did differ by BMI status (*P* < 0.001). Post hoc analysis showed that the effect was underpinned by significant differences in the concentrations of Arg, Asn, Asp, Cit, Glu, Gly, Ser, and Tyr, with high BMI status associated with lower concentrations of Arg, Asn, Gly, and Ser. There was no effect of meal group or BMI on Pre concentrations of the pooled classes of AAs.

**FIGURE 2:**
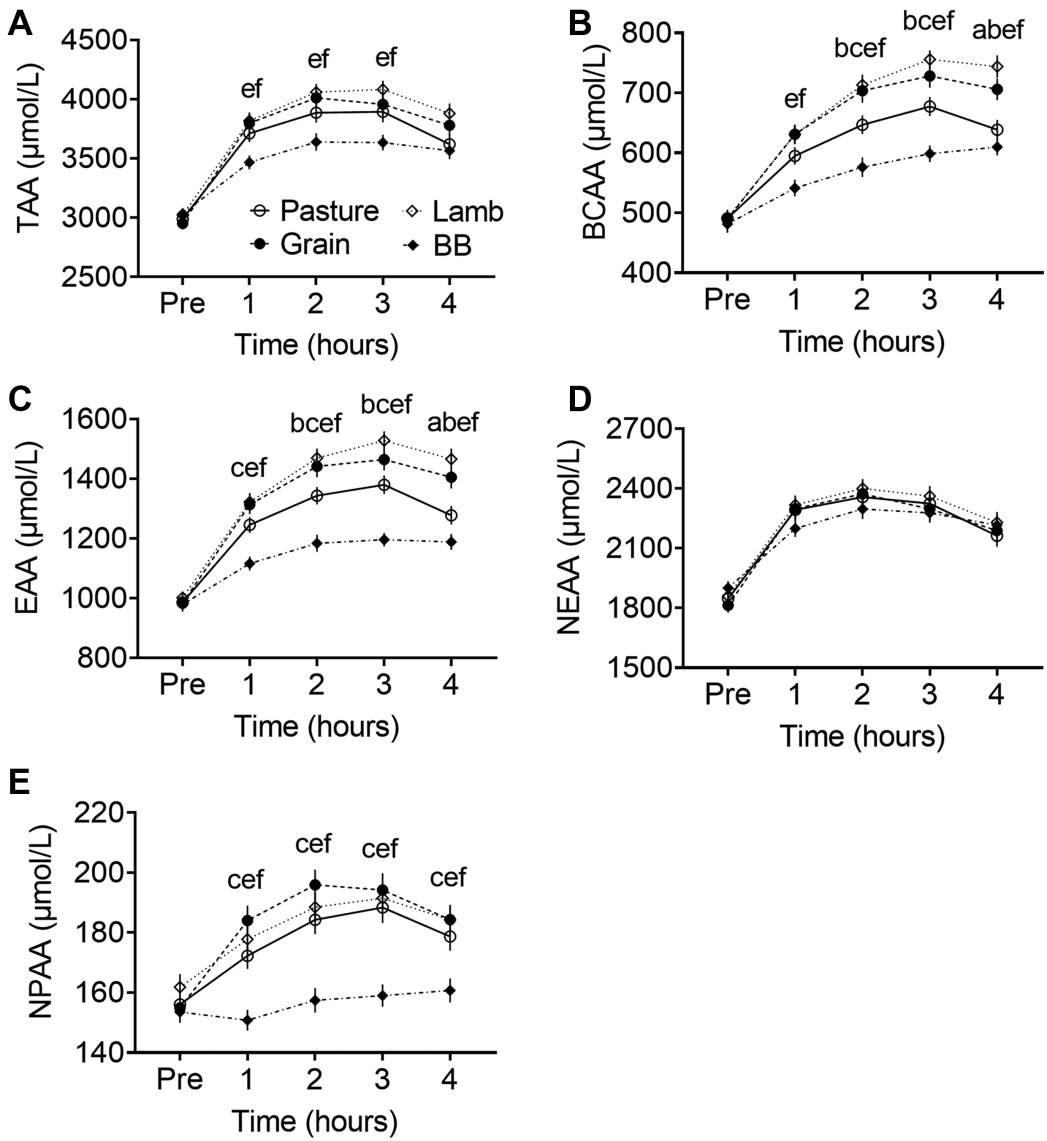
Postprandial plasma concentrations of pooled amino acids. (A) TAAs. (B) BCAAs. (C) EAAs. (D) NEAAs. (E) NPAAs. Markers indicate means ± SEMs (*n* = 29); note the difference in vertical scales. The test meal groups contained either pasture-raised beef (Pasture), grain-finished beef (Grain), pasture-raised lamb (Lamb), or Beyond Burger (BB; Beyond Meat). A significant effect of time was observed for all pooled classes, and interactions with meal groups occurred for TAAs, BCAAs, EAAs, and NPAAs. Post hoc pairwise comparisons by Tukey's test (*P *< 0.05) are as follows: ^a^Between Pasture and Grain, ^b^Between Pasture and Lamb, ^c^Between Pasture and BB, ^d^Between Grain and Lamb, ^e^Between Grain and BB, ^f^Between Lamb and BB. BCAA, branched-chain amino acid; EAA, essential amino acid; NEAA, nonessential amino acid; NPAA, non-proteogenic amino acid; TAA, total amino acid.

Plasma AA responses averaged across individuals within meal groups are shown in **Figure 2**. NEAAs comprised the greatest proportion of circulating TAAs while NPAA was a minor contribution at approximately 5% of the total. Across all the groups, peak concentrations of TAAs occurred near 2 h, driven by the dominating influence of NEAAs, whereas EAAs peaked near 3 h due largely to their BCAA subset. There was very little response in NPAAs for BB, which is consistent with the plant-based, collagen-free composition of that meal. Plasma concentrations of TAAs, BCAAs, EAAs, and NPAAs were lower for BB than the red meats at all postprandial time points, often significantly (*P* < 0.05).

**Table 5** shows the Pre-adjusted AUC for individuals and classes of plasma AAs. The AUC values were positive, indicating net gain in concentration post-meal, except for some negative values among NPAAs and a surprising net loss of methionine in BB. A strong interaction between all AA responses and type of meal was found, which implies a treatment effect (*F* = 5.436, *P* < 0.001). A significant effect of the meal group was then observed for most responses, with BB often the lowest value among the meals. For example, the AUC for EAAs was 133% greater in Lamb, 123% greater in Grain, and 75% greater in Pasture when compared with BB (*P *< 0.05). This was a recurring trend whereby Lamb had the numerically greatest AUC, followed by Grain beef, then Pasture beef. The pattern held for Ile, Leu, Val, Lys, Met, Thr, Trp, Arg, Glu, Pro, Tyr, Cit, BCAAs, EAAs, and TAAs. In contrast, no significant differences between the red meat groups were found for NEAAs and TAAs.

**TABLE 5:** Pre-adjusted AUC for plasma amino acid concentrations over 4 h following the meals^1^

Amino acids	Pasture	Grain	Lamb	BB	P*	Post hoc
EAAs						
Isoleucine	120 ± 9	163 ± 12	180 ± 10	87 ± 7	0.001	abef
Leucine	191 ± 15	261 ± 18	267 ± 14	112 ± 10	0.001	abcef
Valine	209 ± 16	277 ± 19	306 ± 15	137 ± 12	0.001	abcef
Histidine	48 ± 4	54 ± 4	53 ± 5	26 ± 4	0.001	cef
Lysine	237 ± 16	286 ± 16	291 ± 12	115 ± 11	0.001	bcef
Methionine	42 ± 3	56 ± 4	58 ± 4	−8 ± 2	0.001	abcef
Phenylalanine	67 ± 5	84 ± 5	82 ± 6	76 ± 4	0.079	
Threonine	126 ± 10	151 ± 13	157 ± 12	54 ± 8	0.001	cef
Tryptophan	118 ± 8	148 ± 10	152 ± 11	64 ± 10	0.001	cef
NEAAs						
Alanine	540 ± 28	575 ± 44	568 ± 49	387 ± 37	0.004	cef
Arginine	95 ± 7	107 ± 8	118 ± 7	92 ± 7	0.058	
Asparagine	53 ± 5	65 ± 6	60 ± 5	67 ± 5	0.299	
Aspartic acid	2 ± 1	4 ± 1	3 ± 1	0.04 ± 0.5	0.010	e
Glutamic acid	3 ± 7	9 ± 9	13 ± 9	2 ± 6	0.722	
Glutamine	244 ± 33	285 ± 29	256 ± 28	230 ± 38	0.672	
Glycine	155 ± 12	132 ± 17	123 ± 14	68 ± 13	0.001	cef
Proline	378 ± 16	384 ± 17	389 ± 16	290 ± 16	0.001	cef
Serine	72 ± 9	85 ± 11	74 ± 8	60 ± 6	0.251	
Tyrosine	51 ± 5	68 ± 5	76 ± 5	36 ± 5	0.001	bef
NPAAs						
Citrulline	–14 ± 2	-2 ± 2	9 ± 2	–11 ± 3	0.001	abdef
Hydroxyproline	29 ± 1	14 ± 1	11 ± 1	–2 ± 1	0.001	abcef
Ornithine	54 ± 5	57 ± 5	52 ± 4	51 ± 4	0.826	
Taurine	19 ± 3	56 ± 5	11 ± 4	–29 ± 3	0.001	acdef
BCAAs	520 ± 40	702 ± 48	753 ± 38	336 ± 28	0.001	abcef
EAAs	1158 ± 78	1481 ± 88	1546 ± 72	663 ± 57	0.001	abcef
NEAAs	1601 ± 97	1706 ± 113	1700 ± 101	1218 ± 104	0.004	ef
NPAAs	88 ± 8	124 ± 9	84 ± 7	10 ± 6	0.001	acdef
TAAs	2845 ± 176	3299 ± 195	3366 ± 154	1899 ± 157	0.001	cef

1 Values are means ± SEMs in µmol ⋅ h/L; *n *= 29. The test meal groups contained either pasture-raised beef (Pasture), grain-finished beef (Grain), pasture-raised lamb (Lamb), or BB. *P values from the overall *F* ratio test. Post hoc pairwise comparisons by Tukey's test (*P *< 0.05) are as follows: ^a^between Pasture and Grain, ^b^between Pasture and Lamb, ^c^between Pasture and BB, ^d^between Grain and Lamb, ^e^between Grain and BB, ^f^between Lamb and BB. BB, Beyond Burger (Beyond Meat); BCAA, branched-chain amino acid; EAA, essential amino acid; NEAA, nonessential amino acid; NPAA, non-proteogenic amino acid; TAA, total amino acid.

Pasture versus Grain origins of the beef meal significantly affected participants’ responses for only Isoleucine, Leucine, Valine, Methionine, BCAAs, and EAAs.

An example of the variation observed in per-participant responses to meals is shown in **Supplemental Figure 1**. For TAAs, BCAAs, EAAs, and NEAAs the response curves in BB tended to be more tightly clustered than in the other meals. That may reflect the controlled, manufactured composition of the PBMA. Within a meal, relative differences in concentration at Pre between participants tended to remain throughout the test period.

### Plasma glycemic and hormone response

The Pre concentrations of plasma glucose did not differ by meal group but were affected by BMI status. They were higher in participants with a high BMI compared with a normal BMI (5.09 vs. 4.88 mmol/L; *P* < 0.001) and remained higher postprandially across all the meal groups (e.g., a difference in means at 2 h of +0.48 mmol/L; *P* = 0.004).

**FIGURE 3:**
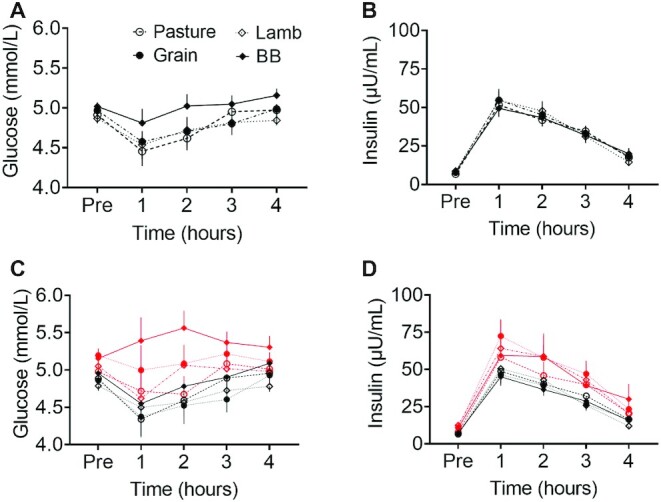
Postprandial plasma concentrations of (A) glucose (mmol/L, *n *= 29); (B) insulin (µU/mL, *n *= 29), (C) separating glucose (mmol/L) responses and (D) insulin (µU/mL) according to normal weight [BMI (kg/m^2^) <25; black lines, *n *= 19] and overweight (BMI >25; red lines, *n *= 10) BMI status of participants. The test meal groups contained either pasture-raised beef (Pasture), grain-finished beef (Grain), pasture-raised lamb (Lamb), or Beyond Burger (BB; Beyond Meat). There was no evidence of a group effect in either metabolite, as the group × time interactions were not significant. Markers indicate means ± SEMs.

The average glucose concentration for BB was numerically highest of all the groups at Pre and remained consistently above the meats postprandially (**Figure 3**). Despite this, there was no statistical evidence of a meal effect, as the group × time interaction was not significant (*F* = 0.523, *P* = 0.90).

The Pre concentrations of plasma insulin were low, which indicates that participants had adequately fasted for each clinic visit. However, those concentrations differed by BMI status. They were higher in participants with a high BMI compared with a normal BMI (10.38 µU/mL vs. 6.75; *P* < 0.001) and remained consistently higher across all groups (e.g., a difference in means at 2 h of +15.98 µU/mL; *P* = 0.016).

Average insulin concentrations increased immediately after eating, then declined steadily over the next 3 h, without a significant difference between groups (*F* = 0.26, *P *= 0.85) or interaction of group × time (*F* = 0.54, *P* = 0.89).

### Appetite assessment

**FIGURE 4:**
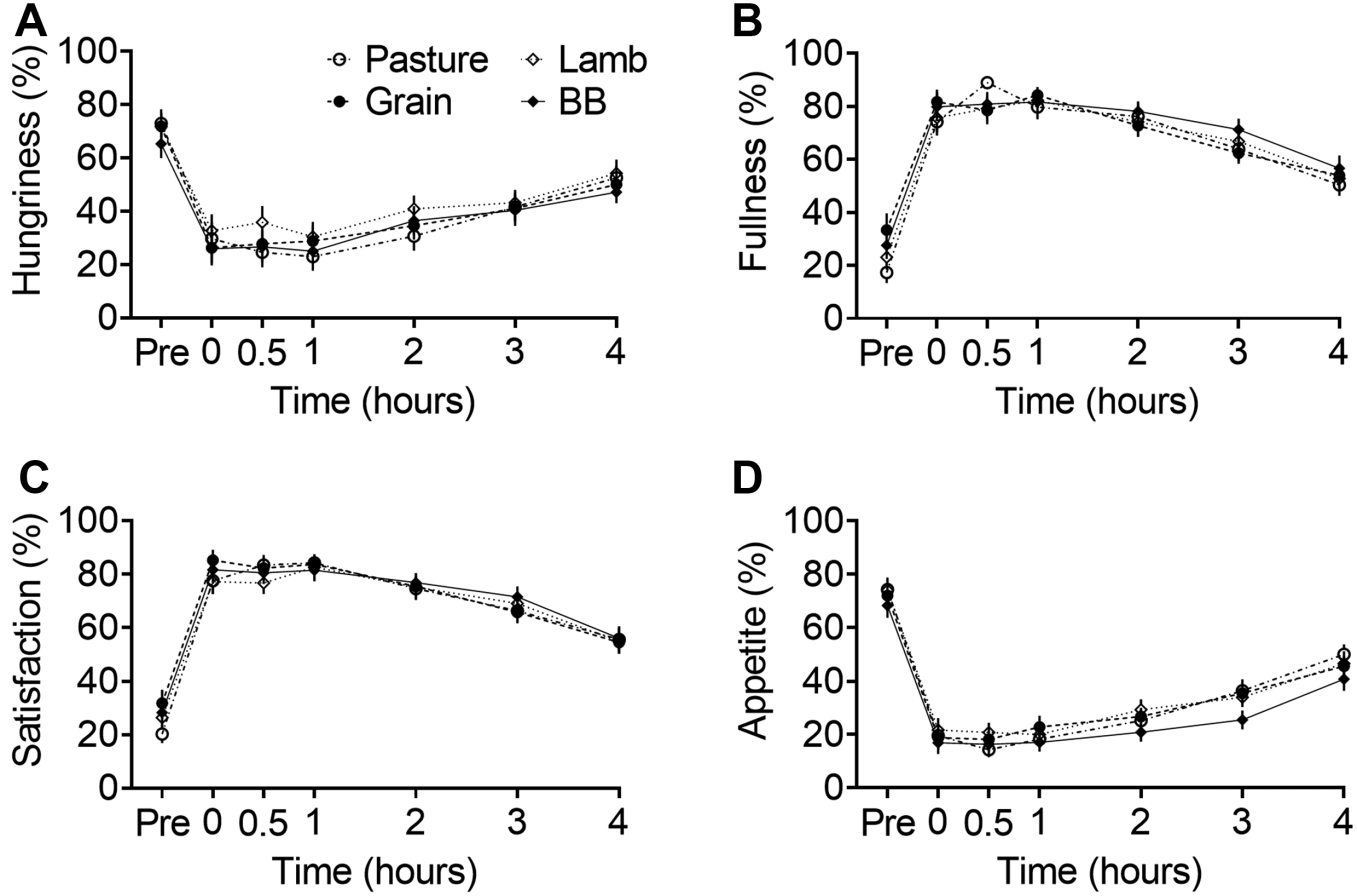
Self-assessed scoring of appetite status anchored at 0 “not at all” and 100 “a lot.” (A) Hungriness score in response to “How hungry do you feel?” (B) Fullness score in response to “How full do you feel?” (C) Satisfaction score in response to “How satisfied do you feel?” (D) Appetite score in response to “How much do you think you can eat?” Markers indicate means ± SEMs (*n *= 29). The test meal groups contained either pasture-raised beef (Pasture), grain-finished beef (Grain), pasture-raised lamb (Lamb), or Beyond Burger (BB; Beyond Meat). There was an effect of time on all qualities of status, but no group × time interactions.

Self-assessed scores for hunger, fullness, satisfaction, and appetite changed significantly from pre-meal to immediately post-meal, then much more slowly over the next 4 h (**Figure 4**). No significant differences were found in these appetite scores between groups, nor did the type of meal consumed affect the desire to eat sweet, salty, savory, or salty food (**Supplemental Figure 2**).

## Discussion

In this study, 29 young men ate a standardized meal containing pasture-raised beef (Pasture), grain-finished beef (Grain), lamb (Lamb), or a PMBA (BB), followed by the measurement of plasma AAs and appetite responses over 4 h. The composition of the meals and the background diet of the participants were also assessed. Concentrations of plasma TAAs, BCAAs, EAAs, and NPAAs were significantly lower following the BB meal compared with the red meat meals. The postprandial AA profiles were consistent with data reported for beef meals in young men (36, 37) and old men (38), whereas a response to PBMAs has not been previously described. Food producers may claim nutrient equivalence between PBMAs and meat, but our results indicate that the protein content of this exemplar PBMA is less able to increase plasma AA availability.

The quality of dietary protein sources is defined in part by a capacity to provide EAAs that cannot be synthesized de novo (39). Given that the EAA content of BB was slightly less than other meats (7–28%), a lower postprandial plasma response could be expected. Despite 7% less content compared with the Pasture meal, we observed a 15% lower plasma AUC, which implies that utilization was attenuated. Emerging evidence has revealed that different dietary protein sources, or food contexts, impact digestion and absorption kinetics of plasma AAs (40–42). The EAA response of BB relative to red meat might also be attributed to the presence of anti-nutritional factors in plant protein sources (43, 44).

Leucine, one of the branched-chain EAAs, is valued not only as proteogenic but is also anabolic, serving as a regulator for the postprandial stimulation of muscle protein synthesis (45–47). It has been previously shown that increasing the proportion of leucine in circulating plasma is required for optimal stimulation of whole-body protein synthesis and can mitigate age-related muscle loss (48). Consistent with overall EAA and BCAA profiles, the greatest postprandial plasma leucine AUC was seen with Lamb, followed by Grain, Pasture, and then BB. Our data suggest that the protein fractional synthesis rate might respond most strongly to a lamb meal compared with other meat types—a scenario that warrants further investigation.

Proline, hydroxyproline, and glycine were also significantly higher in plasma following the meat meals compared with BB. The AAs are constituents of collagen, which establishes the rigid structure of skin, tendon, cartilage, bone, blood vessels, and basement membranes (49). Studies have demonstrated that dietary hydroxyproline can stimulate collagen biosynthesis and is considered important for maintaining the integrity of connective tissues (50). Interestingly, the postprandial responses we observed were proportionally greater than the net content of these AAs, as the red meat meals contained similar proline to BB and only slightly more glycine. Our findings agree with food-composition studies that compared beef with numerous PBMAs and reported little hydroxyproline in the PBMA samples (7).

Differing responses to protein meals could reflect lower digestibility of plant proteins due to the presence of anti-nutritional factors (e.g., trypsin and chymotrypsin inhibitors, phytates, polyphenols) (43), or digestion-resistant networks formed between ingredients (51). Some methods of industrial food processing cause protein degradation and aggregation (52) that render proteins less accessible, but not all are detrimental to nutritional quality (53). Once dietary AAs are absorbed, their utilization is still influenced by the components of the meal. For example, plant carbohydrates stimulate an insulin response, thus upregulating the transmembrane transport of AAs from plasma into muscle (54, 55).

Dietary protein is sometimes associated with satiety, hence our interest in the effect of the test meals on appetite status (56). We found no significant differences in appetite and fullness responses when similar quantities of protein were consumed (48–58 g). This was also reported following beef or soy mixed meals in healthy young men (57) and a clinical study on healthy and overweight women after consumption of mixed meals containing animal proteins (turkey, egg) or plant proteins (58). Taken together, these results suggest that consuming a moderate amount of various protein sources in mixed meals has no effect on the self-assessment of appetite.

The use of a crossover design and the same standardized meal preparation in this study aimed to minimize the intra- and interindividual variability in postprandial AA response. However, we were aware of differences in the food matrix that have been considered a potential factor to influence the digestion rate of proteins. In support, previous work has shown that protein digestion and absorption rates in older men were greater after the consumption of minced beef compared with beef steak (34). Therefore, in the present study, all meat was minced to maximize protein absorption and to mimic the format of the BB product. The mincing also helped to disguise the protein type to participants.

The limitations of this study are acknowledged. First, the participants were all healthy young men. Our results should be carefully considered when applying to more nutritionally vulnerable demographics, especially given the evidence that the elderly have a delayed protein absorption rate (59, 60). Second, we did not measure the postprandial protein fractional synthesis rate; therefore, we cannot identify how the differences in protein absorption kinetics might influence muscle protein metabolism. Having said this, numerous investigations have reported the effects of dietary TAAs and EAAs on fractional synthetic rates. Third, our choice of PBMA was based on the selection criteria outlined in the Methods section. Other products and formulations coming onto the market may be formulated from different sources and amounts of protein and fat, and have different physiological responses. Fourth, the main aim of this study was to provide isocaloric meals with an identical composition of some macronutrients, namely total energy and carbohydrate. While the meals were generally isocaloric, the protein and fat ratios did differ. Lamb, having a lower fat content, concomitantly had a higher protein content. The results presented are relevant to the serving and nutritional composition of meat as it is available to the public; to serve or standardize further is to negate the clinical relevance of any findings. The initial beef and PBMA samples were selected on the basis of being matched for total energy and protein based on information within the food-composition database relevant to the country of study. The actual meat and PBMA samples used in the study differ from those initial estimates, as is common with food-composition variability, which is why the data presented include actual composition analysis of the meals provided. Finally, the slow appearance in plasma of BCAAs from the BB meal makes it ambiguous whether peak BCAA concentration was reached during our postprandial collections. The 4-h duration was based on previous studies on postprandial protein metabolism after a beef meat meal in older men (43, 51) and young men (49).

In the context of wider environmental concerns associated with the production of red meat, the global supply of alternatives has increased substantially (61, 62). However, reliance on plant-based substitutes may have implications for overall diet quality if adopted on a grand scale, acknowledging that the majority of Western diets consume adequate, if not excessive total protein intakes (63). Given findings in the present investigation that demonstrate a protein quality that is unmatched by the plant-based alternative, a balanced approach of moderation and diversity in dietary protein types is warranted.

In conclusion, while meat analogs are designed to mimic the taste, smell, texture, and nutrition of meat, our results show that the bioavailability of their protein is lower compared with red meat. The exemplar PBMA used in this study demonstrated lower postprandial AA delivery into plasma compared with beef and lamb. This may be attributed to a differing profile of AAs in the PBMA as well as their lower bioavailability.

## Supplementary Material

nzac082_Supplemental_File

## Data Availability

Data described in the manuscript, codebook, and analytic code will be made available upon request pending application and approval.

## Notes

This research was funded by the New Zealand Ministry of Business, Innovation, and Employment National Science Challenge High-Value Nutrition; the Meat Industry Association Innovation Limited (a subsidiary of the New Zealand Meat Industry Association); and Beef and Lamb New Zealand Limited. TP is supported by a Heart Foundation Research Fellowship Grant (#1869).

Author disclosures: The authors report no conflicts of interest. The funders were not involved in the data collection, analysis, and interpretation; in the writing of the report, or in the decision to submit the paper for publication.

Supplemental Figures 1 and 2 are available from the “Supplementary data” link in the online posting of the article and from the same link in the online table of contents at https://academic.oup.com/cdn/.

Abbreviations used: AA, amino acid; BB, Beyond Burger; BCAA, branched-chain amino acid; EAA, essential amino acid; NEAA, nonessential amino acid; NPAA, non-proteogenic amino acid; PBMA, plant-based meat analog; TAA, total amino acid; UPLC, ultra-performance liquid chromatography.
