# Supplementary material for: Plasma Amino Acid Appearance and Status of Appetite Following a Single Meal of Red Meat or a Plant-Based Meat Analog: A Randomized Crossover Clinical Trial
Source: Curr Dev Nutr. 2022 May 4;6(5):nzac082. doi: 10.1093/cdn/nzac082 (PMC9154224; doi:10.1093/cdn/nzac082)
Supplement: nzac082_Supplemental_File [file nzac082_supplemental_file.docx]

**Plasma amino acid appearance and status of appetite following a single meal of red meat or a plant-based meat analog: a randomized crossover clinical trial; Toan Pham; Online Supplementary Material**

**Supplementary Figure 1: Amino acid variation in per-participant responses to meals**
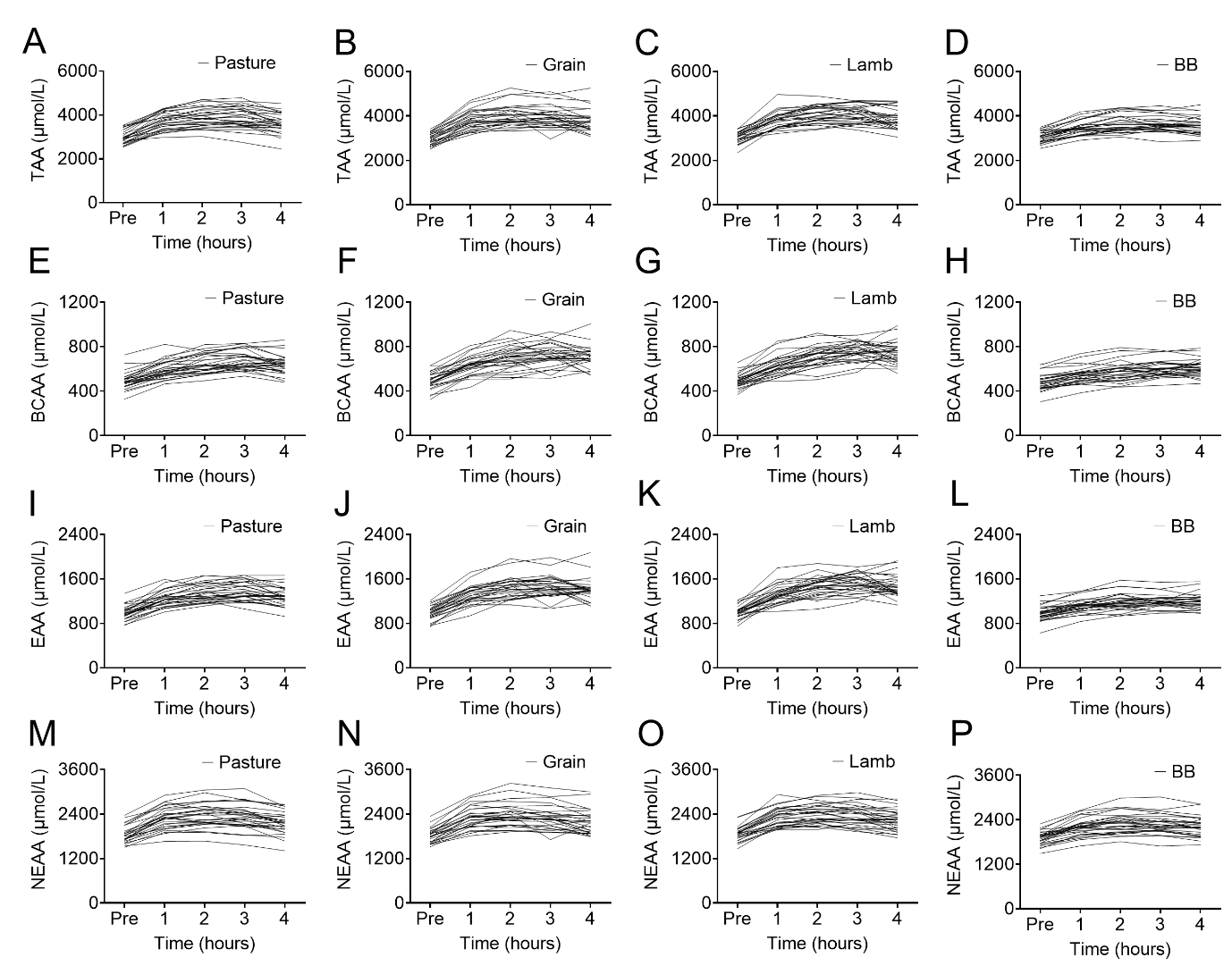


The test meal groups contained either pasture-raised beef (Pasture), grain-finished beef (Grain), pasture-raised lamb (Lamb), or Beyond Burger (BB). n = 29.

**Supplementary Figure 2: Self-assessed scoring of the desire to eat status**


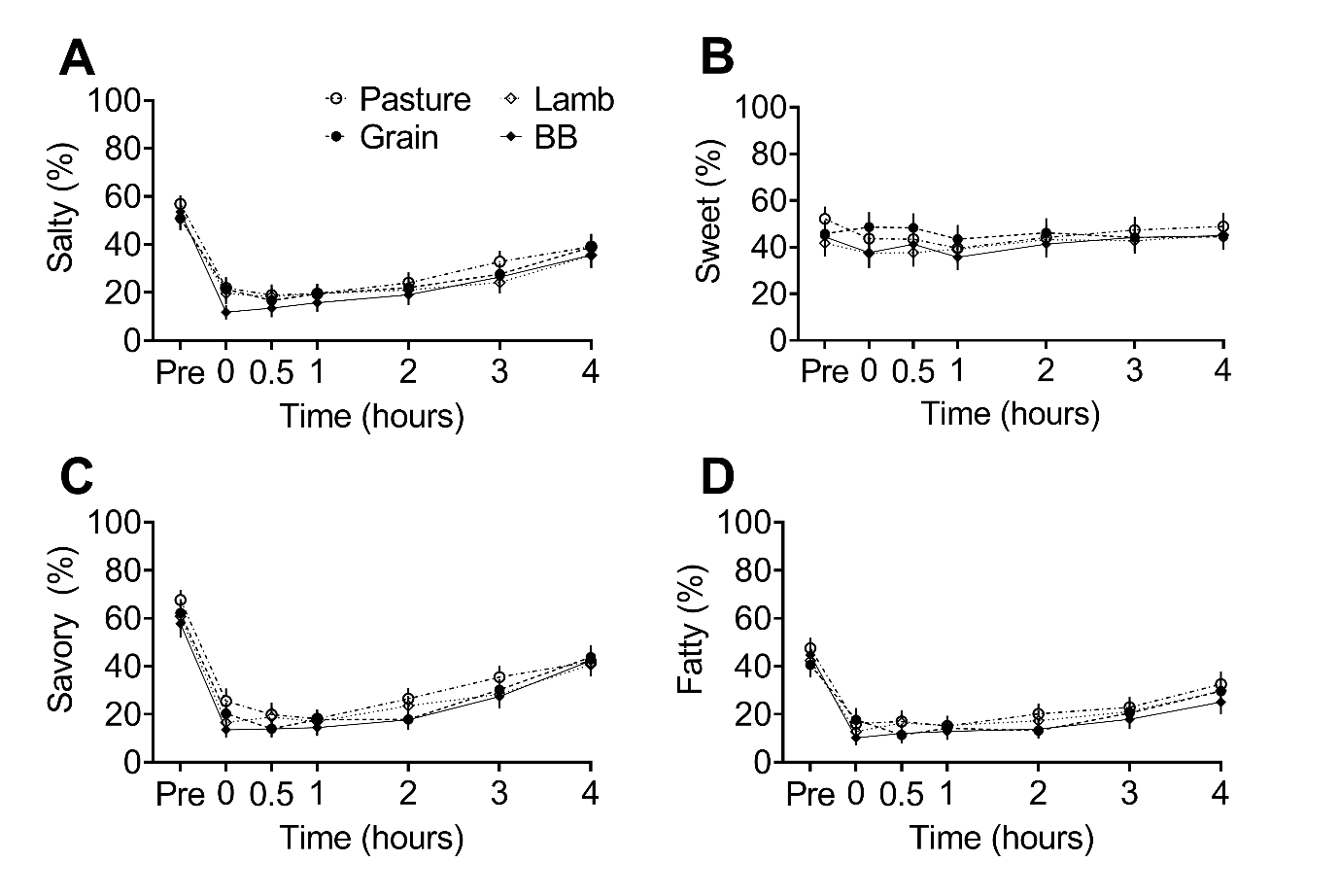


At 0 ‘not at all’ and 100 ‘a lot’. (A) Salty score in response to ‘Would you like to eat something salty?’. (B) Sweet score in response to ‘Would you like to eat something sweet?’. (C) Savory score in response to ‘Would you like to eat something savory?’. (D) Fatty score in response to ‘Would you like to eat something fatty?’. Markers indicate mean ± SEM (n = 29). The test meal groups contained either pasture-raised beef (Pasture), grain-finished beef (Grain), pasture-raised lamb (Lamb), or Beyond Burger (BB).
